# Sokodu, a New Disease

**Published:** 1914-02

**Authors:** 


Sokodu, a New Disease. Translated by Joseph Cuneo, M. D., Denver *Med. Times,* December, 1913. This is a malady which was until recently unknown in Europe, although pretty well generalized in China and Japan. The first victim of sokodu in Europe was an Italian peasant of Tuscany, who had never been outside of his own county. How did he contract the disease? The means by which this infirmity is transmitted, as is the case in bubonic plague, is principally the rats, with the difference that the transmission of sokodu by said rodents is more difficult than in the case of bubonic plague. While that terrible malady can be transmitted by contact alone with rats infected with the disease, or by the simple bite of fleas which have fed on the blood of the rats, it has hitherto seemed necessary, if men contract sokodu, that they be bitten by an infected rat. Therefore the question, "can sokodu develop in human beings in any way other than from rat bites?" European doctors who have deeply studied the malady in the Extreme Orient, do not unanimously agree upon this point. Some of them affirm that the rats are the real cause of the contagion, others claim that the infection can be transmitted from one human being to another. One thing sure is the fact that in China, there are persons affected with sokodu who have never been bitten by a rat. This argument would seem conclusive with regard to that point in the transmission of the disease. It was proven that the Italian peasant had been bitten by a rat. The supposition is that the animal which caused the disease must have been on board of some ship coming from the extreme Orient. Without any loss of time the most rigorous sanitary measures were taken, and a great number of rats were killed in the Italian community where the first case of sokodu occurred, and the spread of the disease was prevented.

Everything seems to indicate that the malady is caused by a micro-organism, but up to the present time it has been impossible to find it, either in the lesion produced in the patient by the bite from an infected animal, or in the patient's blood.

On or about the fifteenth day after infection the patient is taken with chills, fever, headache and vomiting; later the fever becomes intermittent, and the body is covered with very deep pustules, causing intense pain; the pustules may disappear, or appear again at various periods of time; in some cases this phenomenon occurs during a period of years. Sokodu is a malady very difficult to cure. When the disease is in the acute stage, there are very painful cramps in the legs, and disorders of vision. As a constant sequel of sokodu we find conjunctivitis in every case, with the result of a very marked alteration of the eyeball.

The plagues of Asia and Africa, with which the Europeans come every day into contact, on account of their ungovernable lust for commercial expansion or political domination, are slowly disseminating themselves throughout all the European nations. First the sleeping sickness; now will follow sokodu, and after this disease will follow others which at present seem confined to those countries which are awakening the greed of the European nations.

This transmission of disease is like a revenge which the peoples of Asia and Africa are taking against the Europeans, who, under the pretext of civilizing them, are unlawfully encroaching upon their rights and are infamously appropriating their possessions. —*Vida Nueva,* Havana, August, 1913.

